# 

**DOI:** 10.4103/0974-7788.64393

**Published:** 2010

**Authors:** Urmila Thatte

**Affiliations:** *Professor and Head, Department of Clinical Pharmacology, GSMC and KEM Hospital, Parel, Mumbai, India E-mail: urmilathatte@kem.edu*

The response to the first issue of *International Journal of Ayurveda Research* (*IJAR*) has been outstanding. At the time of going to press, we have 70 articles under process (which, at the fifth month of the year, is already as much we got in the whole of last year). More importantly, an article has been cited in another article and the letters to editor received from all parts of the world are proofs of the readership and interest generated by the Journal.

The scholarly guest editorial from Dr. RH Singh in this issue makes an important plea for developing novel research methods in Ayurveda. Research, as we all know (in whichever stream of medicine we are talking of) may be empirical (based on observations and experience), theoretical (based on theory and abstraction) or based on objectives (basic where the search for knowledge is without a defined goal of utility or specific purpose or applied, which is problem-oriented, and directed toward the solution of an existing problem). All types of research are important for expanding the body of scientific knowledge.

The first step in doing any research is formulating an appropriate research question. Light *et al* [1] have said, "Well crafted questions guide the systematic planning of research. Formulating your questions precisely enables you to design a study with a good chance of answering them." In his editorial,[2] Dr. Singh has so beautifully illustrated the problems of generating short-sighted questions while performing research in Ayurveda. I would like to reiterate this with the plea that better research questions should be developed with a multi-disciplinarian approach and an excellent rapport between scientists from both Ayurveda and modern science – but this coming together of the minds has to happen at the outset when formulating a question. Just like when talking with computer experts or statisticians, it is important for a scientist to be able understand their language, scientists from Ayurveda and modern medicine must understand each other. Plucking ideas from imagination without adequate discussions between the two is a sure recipe for disaster. It is this research question that guides the choice of the study design as well as the variables and outcomes of the study.

This issue has an article on study designs. However, I am in complete agreement with Prof. Singh's recommendation that there is an urgent need to develop new study designs to fit in with the principles of Ayurveda. Much has been discussed about the challenges of doing randomized controlled trials of Ayurvedic therapies. Yet, we have not adequately focused on simple descriptive non-interventional studies in Ayurveda. The story of AIDS began with the publication of five case reports of *Pneumocystis carinii* pneumonia at three different hospitals in Los Angeles, California.[3] The editorial note with this article is interesting, stating "The fact that these patients were all homosexuals suggests an association between some aspect of a homosexual lifestyle or disease acquired through sexual contact and *Pneumocystis* pneumonia in this population." and "All the above observations suggest the possibility of a cellular-immune dysfunction related to a common exposure that predisposes individuals to opportunistic infections such as pneumocystosis and candidiasis." From such simple case descriptions building up to more analytical and interventional studies, all study designs have their own place in the generation of evidence. The challenge today is to tweak these well-developed study designs to answer well-defined research questions in Ayurveda relevant to today's health care.

The eligibility criteria for inclusion or exclusion of participants for research as well as the end points and efficacy variables of a trial are other crucial issues on which research needs to be done *vis a vis* Ayurveda to reach any firm recommendations. Should *prakriti* be an inclusion criterion? Can we standardize the methods to clinically assess *prakriti* or will newer objective simpler methods become available? Dr. Singh correctly points out in his editorial the need for caution in choosing the variable to assess efficacy of an intervention – and I can only second this by urging more conceptual research in Ayurveda research methods. The bottom line is that we should be developing our own yardsticks, our own methods, for research so as to develop the evidence in this 21st century. And what better medium could be there to kick start this discussion than a platform like the *IJAR*?

In keeping with the philosophy to promote original research in Ayurveda, this second issue of *IJAR* is rich in both experimental and clinical studies evaluating different concepts of Ayurvedic therapies, including some on quality control issues. The report of the aspirin like glycosidic flavonoid in a classical Ayurvedic anti-pyretic medicine is also very interesting.

I wish you all intellectually stimulating reading and thank you all for the support and interest in the journal.
